# Performance Degradations of MISFET-Based Hydrogen Sensors with a Pd-Ta_2_O_5_-SiO_2_-Si Structure During Long-Term Operation

**DOI:** 10.3390/s19081855

**Published:** 2019-04-18

**Authors:** Boris Podlepetsky, Nikolay Samotaev, Marina Nikiforova, Andrew Kovalenko

**Affiliations:** 1 Micro- and nanoelectronics Department, National Research Nuclear University MEPhI (Moscow Engineering Physics Institute), 115409 Moscow, Russia; nnsamotaev@mephi.ru (N.S.); mnikiforova@bk.ru (M.N.); 2 Induko Ltd., 32/2 Seslavinskaia str., 121309 Moscow, Russia; a.kovalenko@induko.ru

**Keywords:** hydrogen sensors, MISFET, Pd-Ta_2_O_5_-SiO_2_-Si, response parameters, hydrogen concentrations, time-dependence response instability

## Abstract

We present the generalized experimental results of performance degradation of hydrogen sensors based on metal-insulator-semiconductor field effect transistor (MISFET)with the structure Pd-Ta_2_O_5_-SiO_2_-Si. The *n*-channel MISFET elements were fabricated on silicon single chips together with temperature sensors and heater-resistors by means of conventional -technology. Two hundred cycles of responses to different hydrogen concentrations were measured during eight weeks using special measuring and temperature stabilization circuitries with a feedback loop based on the chip’s thermo-sensor and heater. We show how the response parameters change during long-term tests of sensors under repeated hydrogen impacts. There were two stages of time-dependent response instability, the degradation of which depends on operating conditions, hydrogen concentrations, and time. To interpret results, we proposed the models, parameters of which were calculated using experimental data. These models can be used to predict performances of MISFET-based gas analysis devices for long-term operation.

## 1. Introduction

There are a lot of hydrogen sensors commercially available or in development: mechanical, acoustic, optical, catalytic, electrochemical, thermal conductivity-, resistance- and work function-based [1]. The hydrogen sensors are being used in devices to detect the places of hydrogen leakage in hydrogen engines, as well as for monitoring of hydrogen concentrations at nuclear power plants and in storage of electric energy batteries to ensure explosion safety. Besides, gas sensors can also be used in devices to monitor oil and gas deposits, as well as to forecast seismic activity and earthquakes by measuring the concentration of hydrogen and methane emitted from the earth. The capacitor and transistor elements based on metal–insulator–semiconductor (MIS) structures possess the best compatibility with the integrated circuit elements. Therefore, such sensitive elements seem promising to develop integrated hydrogen sensors and gas analysis microsystems-on-chip.

The gas sensors based on MIS-capacitors and field-effect transistors (MISFETs) have been studied by many investigators (e.g., [2,3,4,5,6,7,8,9,10,11,12,13,14,15,16,17]). A great contribution to the developments of gas-sensitive MIS devices has been made by the researchers at Linköping University since their first work in 1975 [2]. Their works [3,4,5,7,12,14] described the gas sensitivity mechanisms, kinetic modeling of hydrogen adsorption/absorption in thin films of catalytic metals and formation of hydrogen atom dipoles in metal–SiO_2_ interfaces of MIS sensors. MIS sensors with different gate materials (palladium, platinum and iridium), with dielectric films SiO_2_, Si_3_N_4_–SiO_2_, TiO_2_–SiO_2_ and Ta_2_O_5_–SiO_2_ have been investigated. The semiconductors Si [2,3,4,5,6,7,8,11,12], GaAs [9] and SiC [14] were used in MIS gas sensors to detect low concentrations of the gases H_2_ [2,3,4], NH_3_ [5], H_2_S [12] and CO [14]. The studies have shown that performance characteristics of MISFET-based hydrogen sensors depend on technological parameters [6], electrical modes [15], chip temperature [17] and external factors (other gases, irradiation [16]).

The researchers of two laboratories at National Research Nuclear University MEPhI (Moscow Engineering Physics Institute) have developed and investigated the number of discrete sensors (MIS-capacitors, Pd- and Pt-resistors) and two types of integrated gas sensors with the structures Pd (or Pt)–SiO_2_–Si, Pd/Ti–SiO_2_–Si and Pd (or Pt)–Ta_2_O_5_–SiO_2_–Si. Both types of integrated sensors contained four elements. In one case, these elements were a capacitor and a MISFET with Pd-gate, thermo-sensitive diode element and the test MISFET with an Al-gate (sensor was called as IHS-1). In another case, these elements are the gas sensitive Pd (or Pt)-resistor and MISFET with a Pd (or Pt) gate, heater-resistor and temperature sensor (sensor was called as IHS-2). Work in this area has been going on for about 25 years. The results of studies of the influence of technological factors (types and thicknesses of dielectrics and metal films, methods of their fabrication) on the metrological characteristics of different types of sensors took a long time (2–4 years for each sensor type). The results of the research were published mainly (80 %) in sources of information that are inaccessible to a wide range of researchers (scientific reports, dissertations and diploma projects). Most of the articles are published in Russian journals and conference proceedings. Numerous experiments have demonstrated that with the same technological parameters, MISFETs have the best performance compared to MIS capacitors in IHS-1 and resistors in IHS-2. In addition, the integrated sensors of the second type (IHS-2), containing MISFETs with a Pd–Ta_2_O_5_–SiO_2_–Si-structure, possess the best stability and reproducibility of characteristics [6,11]. Therefore, in recent years we have investigated the characteristics of this MISFET. The performance characteristics of these sensors and effects of chip temperature, electrical mode and irradiation were studied in our previous works [16,17,18,19,20].

In most of these works, sensor characteristics were studied by measurement of a single or a few (3–5) short-term responses to different hydrogen concentrations. However, previous studies have shown that under long-term hydrogen action, the metrological characteristics of the sensors are subject to change [11]. Performance characteristics (sensitivity, speed, errors and stability) are determined by sensor response parameters, the repeatability of which becomes an important characteristic for long-term operation of gas analytical devices. The question arises: how does long-term hydrogen exposure affect response parameters? To answer this question, we have researched the long-term hydrogen effects on MISFET sensors.

The aims of this paper are to generalize data on research of response parameter changes (performance degradation) of MISFET-based sensors on Pd–Ta_2_O_5_–SiO_2_–Si structures for long-term operation and to propose models, taking in to account the performance degradation.

## 2. Materials and Methods

**Figure 1:**
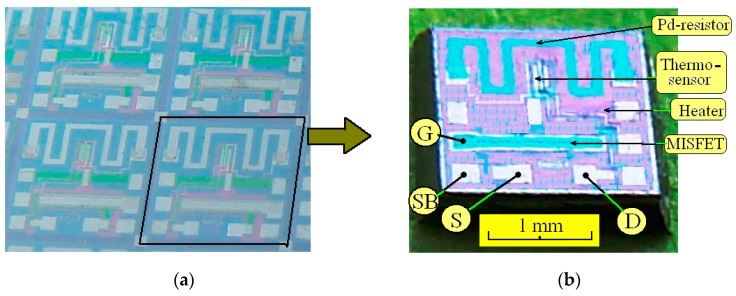
Photos of the sensor chips. (**a**) The fragment of the silicon wafer, undivided into chips; (**b**) The separate chip and designations of the contact pads of the MISFET element: gate (G), source (S), drain (D) and substrate (SB).

**Figure 2:**
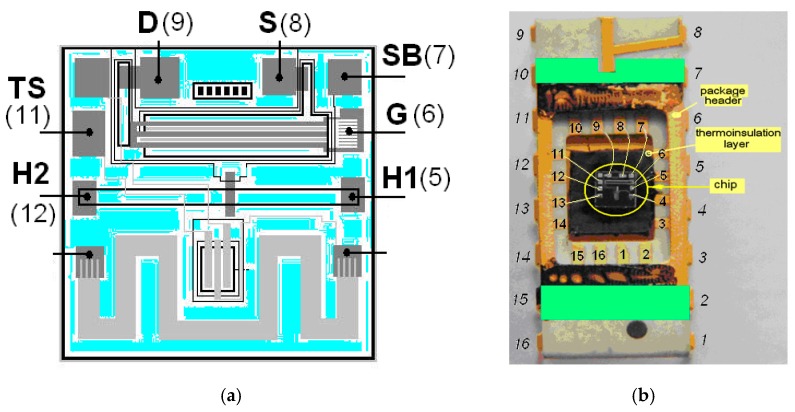
The sensor chip. (**a**) Layout and designations of the contact pads and the pin numbers of elements of a sensor integrated cell: gate (G), source (S), drain (D) and substrate (SB) of Pd–Ta_2_O_5_–SiO_2_–Si FET, the source of Al–SiO_2_–Si FET (TS) and heater-resistor (H1–H2); (**b**) The chip in the sensor housing without cover (numbers are the numbers ouputs’ terminals of the chip).

The testing *n*-channel MISFET element based on a Pd–Ta_2_O_5_–SiO_2_–Si structure was fabricated on a single chip (2 × 2 mm^2^) together with a (*p–n*)-junction temperature sensor and heater-resistor by means of conventional *n*-MOS-technology using laser evaporation Pd-films. Technological processes are presented in detail in [11,17]. Photos of the fragment of the silicon wafer undivided into chips and the separate chip are shown in Figure 1. The structure of the sensor chip and its position in the package is presented in Figure 2. The source *p–n*-junction of the Al-gate MISFET was used as the temperature sensor element. The resistor heating element was used to maintain the operating chip temperature. In this paper, the Pd-resistor was not investigated because it was previously shown that it has worse characteristics than MISFET.

To measure the hydrogen concentration by MISFET, the transistor is embedded in the device’s measuring circuits (signal conditioning circuits). Typically, gas-sensitive MISFETs are applied in analogue signal conditioning circuits. The informative parameter of the volt-metric analogue circuits is the output voltage, *V*. The informative parameter of the MISFET is threshold voltage, *V_T_*. There are three basic electrical modes for those circuits [19]: (1) the measurand *V* is drain bias *V_D_* vs. drain current *I_D_* (*V_T_*) at the constant gate bias *V_G_*; (2) the measurand *V* is *V_G_* vs. *V_T_* at the constant *I_D_* and *V_D_*; 3) the measurand *V* is *V_D_* vs. *V_T_* at the constant *I_D_* and *V_G_*.

The general hydrogen sensitivity can be presented as *S* = |d*V*/d*C*| being equal to |*S_C_* × *S_T_*|, where the circuit’s sensitivity *S_C_* is |d*V/*d*V_T_*|, and *S_T_* = d*V_T_/*d*C* is threshold voltage’s hydrogen sensitivity of the MISFET. The third type of electrical mode can be realized only if value *V_D_* is less than (*V_G_* − *V_T_*); herewith the circuit sensitivity *S_C_* is always less than 0.3, while for the first and second modes, sensitivities *S_C_* ≥ 1.0. Besides, to realize the third type of mode, we need to use the high stability current source, complicating the circuit. That is why in practice, we use either the first mode, when the parameters of the MISFET conversion function are already known, and we can set the optimal gate bias, or the second mode, when we need to investigate the characteristics of new types of MISFET sensors. Therefore, in this work, we used the second mode, in which the change Δ*V* is equal to the change of the threshold voltage Δ*V_T_*.

**Figure 3:**
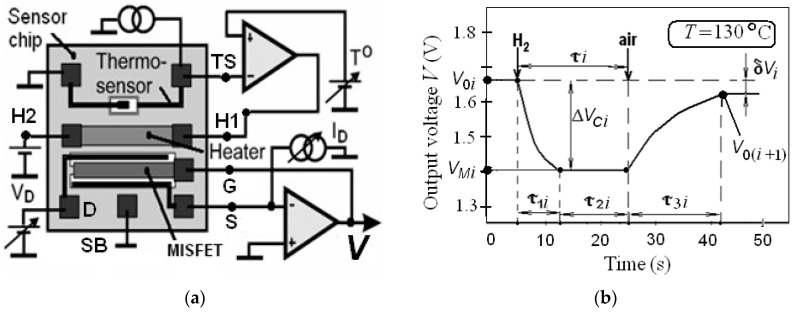
(**a**)The simplified structure of the sensor characteristic measuring circuitry; (**b**) The typical *i*- hydrogen response and its parameters at *C_i_* being equal to 0.05% vol.

To measure the sensor’s hydrogen responses, we used the special circuitry shown in Figure 3a. The circuit provides the constant drain current *I_D_* = 0.1 mA and source-drain voltage *V_D_* = 0.2 V. In this circuitry, the voltage *V* is equal to the gate voltage *V_G_*. The constant chip temperature *T* being equal to 130 ± 2 °C was supported by the temperature stabilization circuitry with feedback loop using the on-chip thermo-sensor and heater [17]. The parameters of each *i*-response are demonstrated in Figure 3b. The initial data of the MISFET parameters and used models are presented in Table 1.

## 3. Results

### 3.1. Experimental Technique

After placing the chip into the sensor housing and connecting the pads to terminals, each sensor was inserted into the circuit. Then, at room temperature, the initial output voltage and response amplitude were measured by a short-term pulse of hydrogen at a concentration of about 1% vol. to estimate the hydrogen sensitivity of each sensor. Five samples with similar characteristics were selected for the experiments (dispersion is not more than 10%).

**Figure 4:**
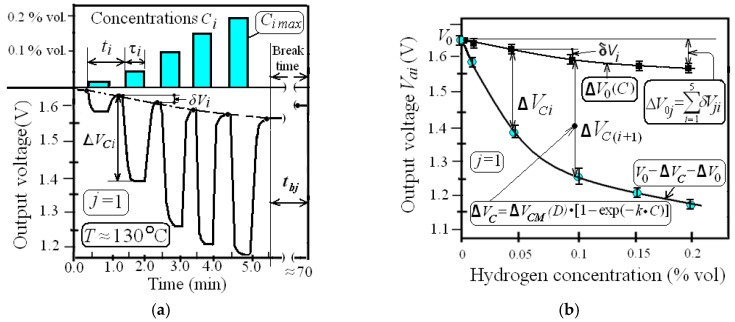
The response parameters: (**a**) Time diagram of the typical *j*-ordinary cycle at different C*_i_*; (**b**) Experimental (symbols) and approximations (lines) of the response residual sum values Δ*V*_0_ and amplitudes Δ*V_C_* vs. concentration *C* for the first *j*-cycle.

Then we tested sensors for eight weeks (five days a week in a row with two-day breaks) by exposure to five repeated hydrogen impacts (*j*-ordinary cycles) per day. In *j*-cycles, each sensor was consecutively exposed to five hydrogen pulses of different concentrations *C_i_* during τ*_i_*(about 30 s) with period *t_i_* about 1 min and a break time *t_bj_* of about 60 min (Figure 4a). The indices *i*, *j*, *k* and *l* are serial numbers: of responses, of ordinary, day and week cycles, respectively. We measured the parameters of each *i*-response of 200*j*-ordinary cycles at hydrogen concentrations of 0.02% vol, 0.05% vol, 0.1% vol, 0.15% vol and 0.2% vol.

Note that each experimental *j*-cycle contains information about the amplitude and time parameters of the ordinary *i*-responses, and also gives the opportunity to calculate the parameters of the conversion function Δ*V_C_* (*C*) and the drift of the initial values of the output voltage Δ*V*_0_ (*C*), so-called “zero-line drift” (ZLD) (Figure 4b). The average values of output voltages *V_ai_*(*C_i_*)and dispersion variation indices ρ*_Vi_* were calculated as:

(1) $$V_{ai}=\frac{1}{N}\cdot \sum_{n=1}^{N}\left|V_{ni}\right|;\;\rho_{Vi}=\frac{1}{N}\cdot \sum_{n=1}^{N}\left|\frac{V_{ni}-V_{ai}}{V_{ai}}\right|,$$

where indices *n* and *N* are respectively serial numbers and quantity of sensors.

Response parameters and their designations are presented in Table 2.

To collect the experimental data, an automated measuring system was used, which measured the voltage *V*(*t*) every second and stored the data in the computer’s memory. The absolute measurement error was 1 mV. The interface board was connected to the data acquisition unit and computer. The hardware and software of the measurement system allows for setting the time steps and range of voltages *V* before each experimental cycle. It is also possible to store experimental data in the form of tables or graphs that can be displayed.

Hydrogen responses were measured approximately five minutes after the sensor was placed in the test chamber and the measurement system was put into operation. The portions of pure hydrogen corresponding to its specified concentrations were injected through a rubber insert in the test chamber lid with a medical syringe. The time of the hydrogen injections did not exceed one second.

### 3.2. Experimental Results

The average values of initial voltage V_0i_, residual values δV_i_, response amplitudes ΔV_Ci_, summary values δV_i_ (designated as ΔV_0_) at different hydrogen concentrations for the first *j*-cycle are demonstrated in Figure 4b. The dependences ΔV_0_(C) and output voltage V(C) are represented by the approximation curves. The same figure shows the analytic models of dependences V(C) and ΔV_C_(C). The average values of parameters of response amplitudes ΔV_Ci_ and sensitivities S_i_ at different C_i_ for first and fiftieth *j*-cycles are presented in Table A1.

The main characteristic that determines the hydrogen sensitivity of the sensor is the conversion function Δ*V_C_*(*C*), the parameters of which are values of Δ*V_CM_*, *k, S_i_* and *S_d_* (Table 2). The speed of the sensor is determined by the time response parameters τ_1*i*_ and τ_3*i*_ (Figure 3b). The repeatability of response parameters (sensor stability) is determined by the parameters Δ*V*_0*S*_ (summary ZLD in Table 2). We have just investigated the changes of these parameters for the long-term operation of MISFETs. Knowing the changes of the parameter *Y* from time *t*, it is possible to estimate the rate *v_Y_* and degree of degradation δ*Y*:

(2) $$v_{Y}=(dY/dt)\;\mathrm{and}\;\delta Y=\{[\int_{0}^{t}v_{Y}(t)dt\;]/Y_{0}\}\cdot 100\%,$$

where *t* and *Y*_0_ are time and primary value of the parameter *Y*, respectively. If the *Y* parameter depends on the total hydrogen dose *D* = ∫*C*(*t*)*dt*, the value *t* should be replaced by *D*. The average values of the responses and the model parameters of conversion function Δ*V_C_*(*C*) for *lkj*-cycles, as well as rates and degree of degradation are demonstrated in Table A2.

**Figure 5:**
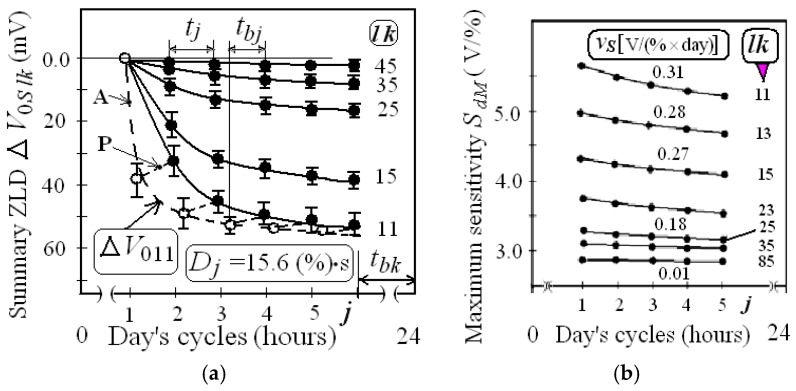
Changes in response parameters during *lk*-cycles on different days: (**a**) Summary ZLD changes, A and P are active and passive ZLD stages; (**b**) Maximum differential sensitivity *S_dM_*.

Note that in each *j*-cycle, hydrogen sensitivity is determined by the integral sensitivity *S_i_* and differential *S_d_*, the values of which are at the maximum at low concentrations and decrease with increasing concentration *C_i_.* The changes of summary ZLD and of maximum sensitivities *S_dM_* during *lk*-cycles on different days are presented in Figure 5a,b.

**Figure 6:**
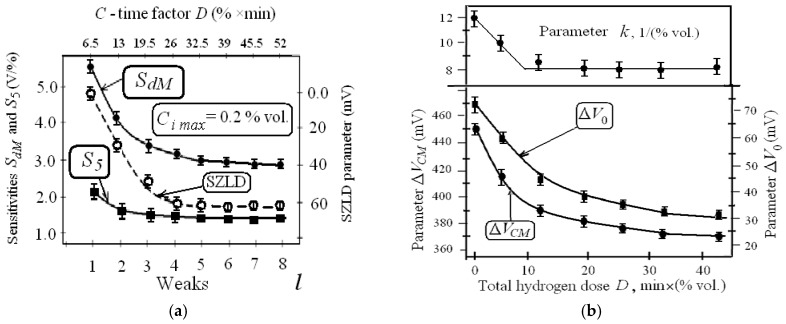
The changes in response parameters as a function of *D*: (**a**) The changes of SZLD parameters, sensitivities *S_dM_* and *S*_5_ during the week’s *l*-cycles; (**b**) Parameters *k* and Δ*V*_0_, maximum response amplitudes Δ*V_CM_* vs. *D*. Symbols correspond to experimental data and lines are approximations.

The changes of SZLD, sensitivities *S_dM_* and *S_5_*, parameters ΔV_CM_ and ΔV_0_ during the eight week cycles are presented in Figure 6a,b.

The analysis of the experimental data (Figure 5 and Figure 6, Table A2) allows us to draw the following conclusions:

1. All parameters of hydrogen responses change as a result of long-term periodic impacts of hydrogen pulses of different concentrations.
2. Immediately after the turning sensors in the operating mode values of output voltage *V* decrease monotonically, reaching a saturation value *V*_0_ being equal to (*V*_00_ − Δ*V*_0*tM*_) after 3–4 min. The average value of Δ*V*_0*tM*_ was equal to about 40 mV. Each hydrogen exposition *j*-cycle started five minutes after the turning sensors in the operating modes (*I_D_* = 0.1 mA, *V_D_* = 0.2 V and *T* = 130 °C). The value of Δ*V*_0*tM*_ changed in the next *j*-cycles within ±5 mV. Such drift of the zero line in time did not depend on the previous hydrogen exposures. The initial values of *V*_0_ of the first responses were equal to about 1.66 V.
3. The changes of initial response voltage of each *i*-response *V*_0*i*_ determined by the parameter δ*V*_0*i*_ decreases with increasing concentration *C_i_* in each *j*-cycle. Then, during the 60 min break time, value *V*_0_ increases by about half of its total change Δ*V*_0*j *_ after hydrogen injections in each *j*-cycle (Figure 4). The relative changes parameter δ*V_i_* being equal to (100% × δ*V*_0*i*_/Δ*V_Ci_*) decreased with increasing *C_i_* within the *j*-cycle. Average values of δ*V_i_* vary in the first *j*-cycle from 25% to 5%, and in the fiftieth *j*-cycle from 15% to 3%.
4. These changes of voltage *V*_0*i*_ were called “zero-line drift” (ZLD). The parameters Δ*V*_0*S*_ (summary ZLD) have positive signs and after repeated actions of hydrogen within four weeks, they are reduced from 50 mV to values less than 5 mV (Figure 5a).
5. The amplitude parameters of the responses Δ*V_Ci_* and maximum amplitude Δ*V_CM_* decrease by 20–40%. The hydrogen sensitivities *S_i_* and *S_dj_* reduced when hydrogen exposition time *t_C_* and dose *D* increase (Table A2).
6. Maximum changes of response parameters (approximately 80%) are manifested in the first stages of hydrogen expositions at *C*-time factor *D* less than 25% vol. × min (see Figure 6).
7. The time parameters of responses decrease slightly by 1–2 s (Table A2).

## 4. Modeling and Discussion

The following model of voltage *V* (*C*,*t*,*t_C_*) was used to interpret the experimental results:

*V* (*C*,*t*,*t_C_*) = *V*_0_ (*D,t*) − Δ*V_C_*(*D,C*), *V*_0_ (*D,t*) = *V*_00_ − Δ*V*_0*t*_(*t*) − Δ*V*_0_*_S_*(*D*); (3)

Δ*V*_0*t*_(*t*) = Δ*V_0t__M_* · [1 − exp(−*t/*τ_0_)] = 0.04 · [1 − exp(−*t/*70)] V, [*t*] is second; (4)

Δ*V*_0_ (*D*) = Δ*V*_0_*_S_*(*D*) = Δ*V*_0_*_SM_* · [1 − exp(−*D*/*D*_0_)] = 0.03 + 0.045 · [exp(−*D*/15)] V; [*D*] is (% vol.) × min;

Δ*V_C_* (*D,C*) = Δ*V_CM_* (*D*) · {1 − exp[− *k*(*D*) × *C*)], *V_CM_* = 0.37 + 0.08 · [exp(−*D*/9)]V; (5)

*k* = (12 − 0.4 · *D*) 1/(% vol.) at *D* < 12 (% vol.) × min, *k* = 8.0 (1/(% vol.)) at *D* ≥ 12 (% vol.) × min.

The value *V*_00_ is a primary voltage of about 1.7 V (at *T* ≈ 130 °C). Formulas (3)–(5) are respectively a general view of the model and the values of the components of the model in general and numerical forms. The values of model parameters Δ*V_0t__M_*, τ_0_, Δ*V*_0_*_SM_*, *D*_0_, Δ*V_CM_* and *k* were determined as parameters of the approximating function of the form Δ*Y*(*x*) = Δ*Y_M_* · (1 − exp(−*νx*)) using experimental data. The parameter Δ*Y_M_* was determined by the maximum value of *Y* by extrapolating the dependence Δ*Y*(*x*) at large *x*, and the parameter *ν* was calculated as:

*ν* = ln {Δ*Y_M_*/[Δ*Y_M_* − *Y*(*x*)]}/*x*, (6)

where values of *x* are *t*, *D* or *C*; values of Δ*Y_M_* are Δ*V_0t__M_*, Δ*V*_0_*_SM_* or *C*; values of *ν* are (1/*τ*_0_) or (1/*D*_0_) or *k*, respectively.

The first component *V*_0_ (*D, t*) of model (3) determines the initial value of voltage *V*. In the measuring circuit (Figure 3a), the voltage *V* is equal to the gate voltage *V_G_*, which depends on the technological and physical parameters of the MISFET specified in Table 1:

*V = V_G_* = φ*_ms_* + φ*_s_* + *a*{φ*_s_* + φ*_T_*exp[(φ*_s_* − 2φ*_s_*_0_)/φ*_T_*]}^0.5^ − [*Q_te_* + *Q_ss_*]/*C*_0_, (7)

where φ*_s_* is the surface potential, which depends on the drain current *I_D_*; *Q_te_* and *Q_ss_* are values of effective charge densities in the dielectric and at the interface SiO_2_–Si. According to (4):

*V*_0_ (*D,t*) = *V*_00_ − Δ*V*_0*t*_(*t*) − Δ*V*_0_*_S_*(*D*), (8)

*V*_00_ = φ*_ms_*_0_ + φ*_s_* + *a*{φ*_s_* + φ*_T_*exp[(φ*_s_* − 2φ*_s_*_0_)/φ*_T_*]}^0.5^ − [*Q_te_*_0_ + *Q_ss_*_0_ (φ*_s_*,φ*_s_*_0_)]/*C*_0_ ≈ 1.7 V

where *Q_te_*_0_ and *Q_ss_*_0_ are initial values of effective charge densities in the dielectric and at the interface SiO_2_–Si.

According to Model (7), the initial transition process Δ*V*_0*t*_ (*t*) after switching the transistor to the operating mode can be explained by the inertia of the chip heating and by the recharge time of the charges *Q_te_* and *Q_ss_*. In previous works [11,17] it is shown that the value of Δ*V*_0*t*_ (*t*) depends on the operating temperature and electrical mode of the MISFET. The dependence of Δ*V*_0*t*_ on the current *I_D_* is due to the dependence of the surface potential φ*_s_*(*I_D_*) on the current, and the temperature dependence Δ*V*_0*t*_ is determined by the temperature dependence of the parameters φ*_T_*(*T*) and φ*_s_*_0_(*T*) (Table 1). The maximum variation of Δ*V*_0*t*_ (*t*) can range from ±10 mV to ±50 mV, and the value of Δ*V*_0*tM*_ is achieved in 2–4 min. The initial time drift of Δ*V*_0*t*_(*t*) is the first type of ZLD.

The second type of ZLD is the change of Δ*V*_0_*_S_*(*D*) according to Model (4) associated with the total hydrogen dose *D* being equal to ∫*C*(*t*)dt, which occurs due to operation of the sensor in a hydrogen environment. Each *j*-cycle has two stages: active (during hydrogen injections; about five minutes) and passive (during break time *t_bj_* ≈ 60 min). In active stage of the *j*-cycle, residual values δ*Vi* decrease and ZLD parameter Δ*V*_0_ increases with increasing *C_i_*, reaching the value Δ*V*_0*j*_ (Figure 4b). Summary ZLD (Δ*V*_0*S**lk**j*_) increases during the day’s cycles; herewith, the rate of increasing *v_SZLD_* being equal to *d*(Δ*V*_0*S*_)*/dt_C_* decreases if hydrogen exposition time *t_C_* and hydrogen dose *D* are rising (Figure 5a). During the passive stages of each *j*-cycle, the values of SZLD decrease, and initial voltage becomes equal to *V*_0 (*j*+1)_. After the fourth week (total number of *j*-cycles exceeds 100), the SZLD reaches its maximum value Δ*V*_0*SM*_ and remains virtually unchanged. According to (4), the parameters of component Δ*V*_0_*_S_* are Δ*V*_0*SM*_ ≈ 45 mV and *D*_0_ ≈ 15% vol. × min.

The observed changes of the initial response voltages *V*_0*i*_ and the phenomenon of ZLD change saturation can be explained by the presence of reversible and irreversible effects in the MIS structure under the hydrogen impacts (in particular, the irreversible formation of stable compounds Pd–H, Pd–O, mechanical stresses and Pd swelling).

Note that the values of Δ*V*_0*t*_(*t*) and Δ*V*_0_*_S_* (*D*) determine the additive errors of “zero” (basic line drift) which do not affect the hydrogen sensitivity and can be compensated for by calibrating the sensor prior to each measurement of the hydrogen concentration.

The second component Δ*V_C_* (*D,C*) of Model (3) can be presented as:

Δ*V_C_* (*D*,*C*) = Δφ*_ms_* (*D*) − Δ*Q_he_*(*D*)/*C*_0_, (9)

where the values of Δφ*_ms_* and Δ*Q_he_*_0_ arethe changes of the Pd–Si work function difference potential φ*_ms_* and of effective charge densities *Q_he_*_0_ at the Pd–Ta_2_ O_5_ interface and in the Ta_2_ O_5_ under the action of hydrogen. The component Δ*V_C_* (*D,C*) determines the hydrogen sensitivities *S_i_* and *S_d_*:

*S_i_* = Δ*V_CM_* (*C_i_,D*) · [1 − exp(−*k* × *C_i_*)]/*C_i_* and *S_d_* = *S_dM_* · exp(−*k* × *C*), (10)

where maximum differential sensitivity *S_dM_* = *k*(*D*) × Δ*V_CM_*(*D*). The dependencies *S*_5_(*D*) and *S_dM_*(*D*) are shown in Figure 6.

Detailed studies [3,4,6,7,8,11,12,15] have demonstrated that the MISFET hydrogen sensitivity depends on several effects which occur in regions of the gas–metal–dielectric structure. Firstly, the hydrogen molecules adsorb on the surface of Pd films and then dissociate into atoms. This dissociation occurs in competition with the adsorption of other ambient molecules, in particular O_2_. In addition to this process, the chemical reactions occur at the (hydrogen + air)–Pd interface (e.g., forming PdO). There is a back reaction between adsorbed hydrogen atoms and adsorbed oxygen atoms, resulting in the formation of water molecules that desorb from Pd at operational temperatures. The hydrogen atom concentration in Pd is proportional to the concentration of adsorbed hydrogen molecules on the surface of Pd and the hydrogen concentration in air. In turn, the concentration of adsorbed hydrogen molecules depends on Pd temperature and the concentrations of other molecules.

Secondly, there is the diffusion of hydrogen atoms through the Pd film to the interface Pd–Ta_2_O_5_, forming a polarized dipole layer of hydrogen atoms. Some hydrogen atoms form the compound Pd–H. The third effect is diffusion and drift of protons in Ta_2_O_5_. All this leads to a change in the effective charge Δ*Q_he_* in Model (9). The equilibrium between the hydrogen concentration at the Pd surface and the hydrogen concentration at the Pd–insulator interface is reached in a few seconds. Besides, there are changes in the palladium structure under the influence of hydrogen and oxygen (formation of Pd–H, Pd–O, mechanical stresses and swelling). So, the density and electrical conductivity of Pd may be changed, as well as the electron work function of Pd, which leads to a change of the parameter Δφ*_ms_* in Model (9). Thus, the hydrogen sensitivity of MIS devices depends on many factors. Simultaneously taking into account all these factors is very difficult or not possible at all.

The parameters Δ*V_CM_*, *k*, *S_dM_* and *S_i_* decrease if total hydrogen dose *D* is rising; herewith, the degradation rates *v_Y_* for the all parameters decrease (Figure 5a and Figure 6, Table A1, Table A2). Such changes can also be explained by the existence of irreversible effects in the (hydrogen + air)–Pd–Ta_2_O_5_ structure that tends to saturation.

It was found that the time parameters of responses depend on hydrogen concentrations; if hydrogen concentration increases, the response times τ_1_ and τ_3_ decrease. For τ_1_, this effect can be explained by increasing the diffusion rate of hydrogen atoms moving from the palladium surface to the Pd–Ta_2_O_5_ interface, where the polarized dipole layer is formed. The diffusion rate increases due to an increase in the concentration gradient of hydrogen atoms in Pd being proportional to the concentration of adsorbed H_2_ molecules on the palladium surface, which is proportional to *C*. The relaxation time τ_3_ is partly determined by the reverse diffusion of hydrogen atoms through the Pd film to the Pd–air interface, the diffusion rate of which is proportional to the concentration of hydrogen atoms in Pd, and accordingly to the concentration of hydrogen molecules *C* in air. As a result of long-term cyclic hydrogen impacts, the time parameters of the responses decrease slightly (by 1–2 s) (Table A2).

It should be noted that the stability and reproducibility requirements of the sensors depend on their application. If the gas sensor is used to detect the presence of gas in the environment, the effects of ZLD and the reduction of hydrogen sensitivity do not affect its operation. In this case, it is enough that the sensor has palpable hydrogen sensitivity. In devices for short-term measurements of gas concentrations, degradation of the amplitude parameters of the responses should be taken into account. Therefore, such devices need to be calibrated before each measurement. This applies to devices used for assessing the degree of fire in mines, one-time measurements of gas concentrations in the environment and medical diagnosis. In devices for long-term measurements of gas concentrations in the environment, it is also necessary to take into account degradation processes. Therefore, after the first calibration, you must periodically calibrate the device or replace the sensors. Such modes of operation relate to fire hazard detectors and devices for environmental monitoring.

Presented in the general and numerical form, the above models can be used to predict the degradation characteristics of sensors based other types of electrical circuits, including circuits with several MISFET sensing elements. In electrical models of other circuits, the informative parameter can be the threshold voltage of the transistor *V_T_*, which for the MISFET studied in this paper, is associated with the output voltage *V* as *V_T_* = (*V* − 0.35) V. Devices based on other circuits may have a higher sensitivity and linearity of the conversion function. However, the degree and rate of degradation of metrological characteristics are determined only by the degradation parameters of MISFET.

## 5. Conclusions

This paper generalized experimental data on research of response parameter changes of MISFET-based sensors on Pd–Ta_2_O_5_–SiO_2_–Si structures undergoing long-term operation, and the proposed models in general and numerical form take into account performance degradations. The analysis of the experimental data allows us to draw the following conclusions.

All parameters of the hydrogen response change as a result of long-term periodic impacts of hydrogen pulses of different concentrations. Three amplitude and two time response parameters were measured. The changes of initial response voltage of each response *V*_0*i*_ decreases with increasing concentration *C_i_* in each hydrogen impact cycle. The hydrogen sensitivity was determined by the conversion function Δ*V_C_*(*C*) based on the approximation of the averaged experimental data Δ*V_C_*(*C_i_*). Changes in response parameters were considered depending on the total hydrogen dose *D* being equal to ∫*C*(*t*)*dt*. When the hydrogen sensitivity is reduced, the hydrogen exposition time and dose *D* increase. As a result of the effects of 200 identical repetitive cycles with different hydrogen concentrations *C_i_*, the amplitude parameters of the responses decrease by 20–40%.

All tested MISFET sensors undergoing long-term operation have degradation features: the reduction of hydrogen sensitivity and “zero-line drift” (ZLD), which depend on operating conditions and accumulated hydrogen dose *D*. The maximum change of response parameters (approximately 80%) are manifested in the first stages of hydrogen expositions at *D* less than 25% vol. × min. The time parameters of responses decrease slightly by 1–2 s. Herewith, the degradation rate of the response parameters decreases with the growth of the total concentration dose *D*.

The observed changes of the response parameters and the phenomenon of ZLD change saturation were explained by the presence of reversible and irreversible effects in the (hydrogen + air)–Pd–Ta_2_O_5_ structure under hydrogen impacts (in particular, the irreversible formation of stable compounds Pd–H, Pd–O, mechanical stresses and Pd swelling). These effects for practical applications of hydrogen sensors were taken into account as the additive errors of “zero” (basic line drift), which can be compensated for by calibrating the sensor prior to each measurement of the hydrogen concentration.

To interpret the experimental results, we proposed models in both general and numerical forms that also allowed for estimating the response parameter degradations effect on hydrogen sensitivity. The models were obtained on the basis of the averaging of experimental data and approximations of dependences of model components on time and hydrogen concentrations. The presented models can be used to predict the degradation characteristics of devices and sensors based on other types of electrical circuits, including circuits with several MISFET sensing elements.

The stability and reproducibility requirements of the sensors depend on their application. If the gas sensor is used to detect the presence of gas in the environment (e.g., to detect the location of hydrogen leakage in a hydrogen engine), the effects of ZLD and the reduction of hydrogen sensitivity do not affect its operation. In this case, it is enough that the sensor has palpable hydrogen sensitivity. In devices for short-term measurements of gas concentrations, degradation of the amplitude parameters of the responses should be taken into account. Therefore, such devices need to be calibrated before each measurement. These include devices for assessing the degree of fire in mines, one-time measurements of gas concentrations in the environment and medical diagnosis. In devices for long-term measurements of gas concentrations in the environment, it is also necessary to take into account degradation processes. Therefore, after the first calibration, we must periodically calibrate the device or replace the sensors. Such modes of operation relate to fire hazard detectors and devices for environmental monitoring.

It should be noted that the preparation of sensors, the experiments and the processing of the data took about a year. Despite the previous studies and the results of this work, the issues of the degree of degradation of sensor characteristics in continuous operation, as well as effect of aging, remain to be explored.

## Figures and Tables

**Table 1:** Average values of the parameters of MISFETs and the used parameter models (3).

Symbols	Parameters	Values
*d*_1_	thickness of Ta_2_O_5_	90–100 nm
*d_2_*	thickness of SiO_2_	80–90 nm
*d*	*d*_1_ + *d_2_*	170–190 nm
ε_0_	vacuum dielectric constant	8.85 × 10^−12^ F/m
ε_1_	relative permittivity of Ta_2_O_5_	25
ε_2_	relative permittivity of SiO_2_	4
ε_3_	relative permittivity of Si	12
ε	value (*d*ε_1_ε_2_)/(ε_1_*d*_2_ + ε_2_*d*_1_)	6.9
*C*_0_	dielectric capacitance (ε_0_ε)/*d*	30 nF/cm^2^
*N_A_*	concentration of acceptors	5 × 10^15^ cm^−3^
*b*	transconductance	2.0 mA/V^2^
*k*	Boltzmann constant	1.38 × 10^−23^ J/K
*T*	operating chip temperature	400 K
*q*	electron charge	1.6 × 10^−19^ C
(*q · *φ*_ms_*_0_)	Pd–Si work functions difference	0.08 eV
φ*_T_*	thermal potential *kT*/*q* for *T* = 400 K	33 mV
φ*_s_*_0_	φ*_T_*ln(*N_A_*/*n_i_*) for *T* = 400 K ≈ 130 °C	0.21 V
*a*	(2*q* · ε_3_ε_0_*N_A_*)^0.5^/*C*_0_	1.33 V^0.5^

**Table 2:** Designations and *n_lkji_*-response parameters (*Y_lkji_*) for the characterization of instabilities and performance degradation parameters of output voltage (*C*,*t*,*t_C_*). ZLD = zero-line drift.

*i*-Response	*j*-Cycle	*k*-and *l*-Cycles
*V*_0*i*_—initial voltage;	maximum amplitude Δ*V_CM_*;	number of days (*n_k_*) and weeks (*n_l_*)
τ*_i_*—H_2_ pulse duration: 25–30 s;	amplitude’ changes: δ*V_CM__j_* = Δ*V_CM_* − Δ*V_CM j_*;	amplitude changes: δ*V_CMlk_* = Δ*V_CM_* − Δ*V_CMlk_*;
*C_i_*—H_2_ concentration;	differential sensitivity: *S_dj_* = *d*(Δ*V_C_*)/*dC*;	total exposition time: *t_C_* = *5* · *n_j_* · *n_k_* · *n_l_* · τ*_i_* ≤ 500 min;
δ*V*_0*i*_—residual value;	maximum sensitivity *S_dMlk_*;	summary ZLD (*j* = 1; *k* = 1): Δ*V*_0*Sk*_ = *V*_0*k*_ − *V*_0(*k*+1)_; Δ*V*_0*Sl*_ = *V*_0*l*_ − *V*_0(*l*+1)_; Δ*V*_0*S*_ = *V*_0111_ − *V*_0(*lkji*) max_;
Δ*V_Ci_*—response amplitude;	break time *t_bj_* ≈ 60 min;	
τ_1*i*_—response time;	cycle time *t_j_* ≈ 75 min;	
τ_3*i*_—relaxation time;		
*t_i_*—response period: ≈1.0 min	ZLD: Δ*V*_0*j*_ = *V*_0*j*1_ − *V*_0*j i*max_;	
*S_i_* = Δ*V_Ci_*/*C_i_*—sensitivity;		*C*-time factor: *D* = *n_k_* · *n_l_* · *D_j max_* ≤ 52 (% × min)
*D_i_* = (*C_i_* · *τ_i_*) is the *C_i_*-time factor	Summary ZLD(SZLD) (*i* = 1): Δ*V*_0*Slkj*_ = *V*_0*lk*1_ − *V*_0l*k(j*+1)_	

## Appendix A

**Table A1:** The average values of response amplitude ΔV_Ci_ and its parameters for the first and fiftieth *j*-cycles.

*C*,% vol.	*j* = 1			Total *j* = 50		
	Δ*V_C_*_1_, mV	ρ*_V_*_1_	*S_i_*_1_,V/%	Δ*V_C_*_2_, mV	ρ*_V_*_2_	*S_i_*_2_,V/%
0.02	82	0.04	4.1	58	0.05	2.9
0.05	262	0.01	5.24	212	0.014	4.24
0.1	344	0.009	3.44	282	0.01	2.82
0.15	355	0.008	2.37	290	0.01	1.88
0.2	415	0.01	2.07	340	0.012	1.7

**Table A2:** Average values of response and model parameters of the conversion function Δ*V_C_* (*C*) for *lkj*-cycles.

↓parameters /*lkj→*	111	155	255	355–455	555–655	755–855	Total Δ*Y_D_*,%
Δ*V_CM_*, V/ρ*V_CM_*, %	0.46/4.3	0.41/3.7	0.39/3.8	0.38/3.9	0.37/4.0	0.36/4.2	21.7
*k*, 1/%/ρ*k*, %	12/8.2	10/8.8	8.5/10.6	8.2/10.6	8.1/11.5	8.0/11.5	33.3
*S_dM_*, V/%/ρ*S_dM_*, %	5.52/12.5	4.1/12.5	3.32/14.4	3.12/14.5	2.96/15.5	2.88/15.7	47.8
*v_S_*, V/(% × day)	-	0.28	0.16	0.024	0.016	0.08	-
*S*_5_, V/%/ρ*S*_5_, %	2.1/2.4	1.8/2.8	1.7/3.1	1.55/3.2	1.50/3.3	1.45/3.45	31.0
Δ*V*_0_*_S__lk__j_*, mV	43	37	16	7	3		-
*v*_Δ_*_V_*_0_, mV/(day)	-	7.4	3.2	1.4	0.6		-
τ_1_*_i_*, s/τ_3_*_i_*, s (*i* = 2)	10/15	9/15	8/15	7/16	7/15		30/6.6
τ_1_*_i_*, s/τ_3_*_i_*, s (*i* = 5)	7/17	7/16	6/16	6/16	7/15		14.3/11.8
*D*, % × min	0.26	6.5	13	19.5–26	32.5–39	45.5–52	-
